# Prediction of heterotrimeric protein complexes by two-phase learning using neighboring kernels

**DOI:** 10.1186/1471-2105-15-S2-S6

**Published:** 2014-01-24

**Authors:** Peiying Ruan, Morihiro Hayashida, Osamu Maruyama, Tatsuya Akutsu

**Affiliations:** 1 Bioinformatics Center, Institute for Chemical Research, Kyoto University, Gokasho, Uji, Kyoto, Japan; 2 Institute of Mathematics for Industry, Kyushu University, 744 Motooka, Nishi-ku, Fukuoka, Japan

## Abstract

**Background:**

Protein complexes play important roles in biological systems such as gene regulatory networks and metabolic pathways. Most methods for predicting protein complexes try to find protein complexes with size more than three. It, however, is known that protein complexes with smaller sizes occupy a large part of whole complexes for several species. In our previous work, we developed a method with several feature space mappings and the domain composition kernel for prediction of heterodimeric protein complexes, which outperforms existing methods.

**Results:**

We propose methods for prediction of heterotrimeric protein complexes by extending techniques in the previous work on the basis of the idea that most heterotrimeric protein complexes are not likely to share the same protein with each other. We make use of the discriminant function in support vector machines (SVMs), and design novel feature space mappings for the second phase. As the second classifier, we examine SVMs and relevance vector machines (RVMs). We perform 10-fold cross-validation computational experiments. The results suggest that our proposed two-phase methods and SVM with the extended features outperform the existing method NWE, which was reported to outperform other existing methods such as MCL, MCODE, DPClus, CMC, COACH, RRW, and PPSampler for prediction of heterotrimeric protein complexes.

**Conclusions:**

We propose two-phase prediction methods with the extended features, the domain composition kernel, SVMs and RVMs. The two-phase method with the extended features and the domain composition kernel using SVM as the second classifier is particularly useful for prediction of heterotrimeric protein complexes.

## Background

To identify a set of proteins as a functional protein complex is essential for understanding molecular systems in living cells. Several proteins form a complex and work as a transcription factor, whereas there exist another type of proteins that work as enzymes. Hence, to identify proteins that constitute such transcription factors is useful for uncovering gene regulatory networks and metabolic pathways. Many computational methods have been developed for predicting protein complexes from protein-protein interaction networks [1,2]. Enright *et al*. developed the Markov cluster (MCL) algorithm [3], which repeatedly executes two operators called expansion and inflation to a matrix whose element represents the transition probability from a protein to another. The expansion operation takes the power of the matrix, and the inflation operation takes the Hadamard power of the matrix. MCL is fast and efficient because of these operations. Macropol *et al*. developed the repeated random walks (RRW) method [4], which iteratively expands a cluster depending on the probabilities in steady states of random walks with restarts. Maruyama and Chihara improved the RRW method by weighting the restart probabilities and proposed the node-weighted expansion (NWE) method [5]. Bader and Hogue developed the molecular complex detection (MCODE) method [6], which uses a modified clustering coefficient defined by edge density in a subset of the original and adjacent vertices to find densely connected regions. King *et al*. developed the restricted neighborhood search clustering (RNSC) method [7], which selects clusters generated by a cost function according to the cluster size, density and functional homogeneity. Altaf-Ul-Amin *et al*. developed DPClus [8], which tries to find densely connected regions. Chua *et al*. developed the protein complex prediction (PCP) method [9], which finds maximal cliques using the functional similarity weight based on indirect interactions. Liu *et al*. developed the clustering based on maximal cliques (CMC) method [10], which generates all maximal cliques from the protein-protein interaction networks, and assembles highly overlapped clusters based on their interconnectivity. Wu *et al*. developed the core-attachment based (COACH) method [11]. Most methods basically focus on finding densely connected subgraph in protein-protein interaction networks. Hence, it is considered to be difficult that they detect small protein complexes because, for instance, the edge density of two interacting proteins is always 1.0 even if the proteins do not form a complex.

However, protein complexes with small sizes occupy a large part of whole known protein complexes. CYC2008 is a comprehensive catalogue of 408 manually curated yeast protein complexes [12]. In the catalogue, 172 complexes (42%) are heterodimeric, and 87 complexes (21%) are heterotrimeric as reported also in [13]. In our previous study, hence, we developed a method using our proposed kernel for predicting heterodimeric protein complexes [14], which outperforms an existing method using the naive Bayes classifier [15]. In this paper, we propose prediction methods for heterotrimeric protein complexes by extending techniques in our previous method on the basis of the idea that heterotrimeric protein complexes are not likely to share the same protein with other heterotrimeric protein complexes. For that purpose, we apply supervised learning methods twice such as support vector machine (SVM) [16] and relevance vector machine (RVM) [17]. Tatsuke and Maruyama developed the proteins' partition sampler (PPSampler) method based on the Metropolis-Hastings algorithm, which generates clusters whose sizes follow a power-law distribution, and outperforms other existing methods in F-measure for whole protein complexes [13]. For prediction of heterotrimeric protein complexes, they reported that the F-measure of NWE was better than those of the existing methods, MCL, MCODE, DPClus, CMC, COACH, RRW, and PPSampler. We perform 10-fold cross-validation, and calculate the average F-measure. The results suggest that our proposed methods outperform the existing method NWE.

## Methods

In this section, we propose prediction methods for heterotrimeric protein complexes. More accurately, we consider the following problem: Given a network of protein-protein interactions weighted by some reliability, determine whether or not three distinct proteins that are connected in the protein-protein interaction network form a protein complex. Let *G*(*V*, *E*) be an undirected graph with a set *V *of vertices and a set *E *of edges, representing the protein-protein interaction network. Here, a vertex represents a protein, an edge (*i*, *j*) represents an interaction between proteins *P_i _*and *P_j_*, and the weight *w_ij _*represents reliability and strength of the interaction between *P_i _*and *P_j_*. In this paper, we use the WI-PHI database [1] as edge weights, which has been calculated from heterogeneous biological experimental data. We call *P_i _*a *neighboring *protein to *P_j _*if (*i*, *j*) ∈ *E*. Then, our proposed methods use the support vector machine (SVM), its discriminant function, and the relevance vector machine (RVM).

### Support and relevance vector machine

We briefly review the support and relevance vector machines [16,17]. Suppose that *N *training data {***x***_*i*_, *t*_*i*_} with target *t_i _*∈ {-1, 1} are given. For our purpose, ***x***_*i *_corresponds to a set of three distinct proteins, *t_i _*= 1 corresponds to the case that the set forms a heterotrimeric protein complex. Then, we consider linear models represented by the form

(1) $$y\left(x\right)=\overset{M}{\underset{i=1}{\sum }}a_{i}\phi_{i}\left(x\right)+b,$$

where *ϕ_i _*denotes a basis function, *M *denotes the number of basis functions, *a_i _*denotes the coefficient, and *b *denotes the bias parameter. In the SVM, *ϕ*_*i*_(***x***) is implicitly defined as *K*(***x***_*i*_, ***x***) with a positive semidefinite kernel function *K*, *M *is equal to *N*, and *a_i _*and *b *are determined by maximizing the margin. New sets ***x ***of proteins are classified according to the sign of *y*(***x***). We make use of this discriminant function *y*(***x***) in our proposed methods.

The RVM is a Bayesian sparse kernel technique for classification and regression, and shares some characteristics of the SVM. As well as the SVM, the basis functions of the RVM are given by kernels, which are not required to be positive semidefinite. It, however, is known that training time of the RVM is in general longer than that of the SVM. In the RVM, a hyperparameter *γ_i _*for each parameter *a_i _*and a prior distribution over parameters *a_i _*are introduced to obtain a sparse model. For the classification, the model in Eq. (1) is transformed as *σ*(*y*(***x***)), where *σ*(*y*) denotes the logistic sigmoid function 1/(1 + *e^-y^*), and *a_i _*and *b *are determined by maximizing the marginal log-likelihood with respect to *γ*.

### Extension of feature space mapping

**Figure 1:**
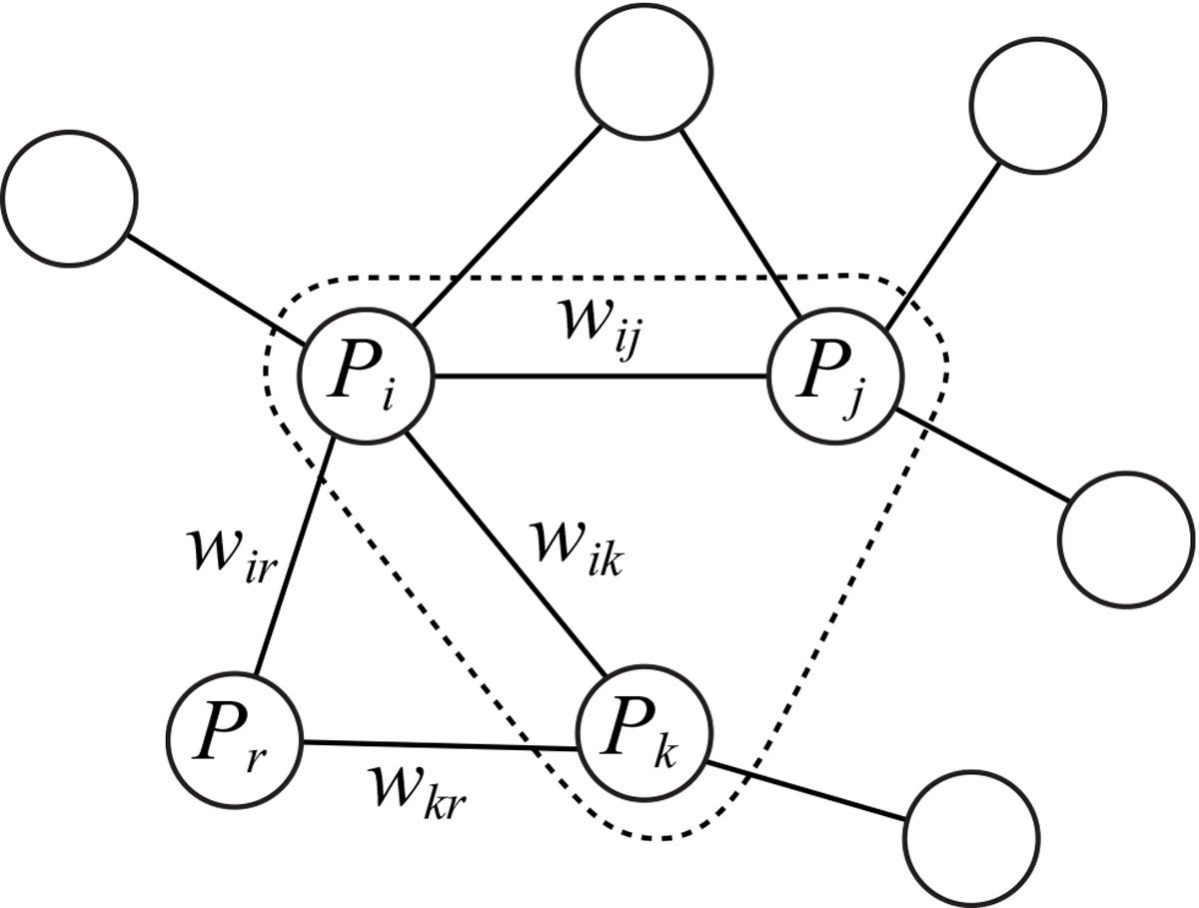
**Example of a subgraph including three focused proteins *P_i_*, *P_j_*, *P_k _*and their neighboring proteins**. In this example, protein *P_r _*is neighboring to both of *P_i _*and *P_k_*.

In our previous study, we proposed seven feature space mappings for prediction of heterodimeric protein complexes [14]. These are based on the idea that the reliability of the interaction in a heterodimer should be high and conversely the reliability of the interaction between a protein in a heterodimer and a protein not in the heterodimer should be low. We extend the feature space mappings for two interacting proteins to mappings for three proteins. Table 1 shows detailed extended mappings for three distinct proteins *P_i_*, *P_j_*, and *P_k _*that are connected in the protein-protein interaction network. Here the fifth mapping in the previous study is eliminated because more neighboring proteins increase the maximum of differences close to the maximum of neighboring weights denoted by (F3). (F1) and (F2) denote the maximum and minimum of the weights of interactions between *P_i_*, *P_j_*, and *P_k_*, respectively. The first feature in the previous study is the weight of the interaction between two proteins. Since there are at least two interactions for three focused proteins and we cannot use all the weights as elements of our feature vector without changes, we take the maximum and minimum of the weights (see Figure 1). In addition, the proteins in a heterotrimer should interact with each other, and (F2), which is the minimum of the weights, is expected to be high. (F3) and (F4) denote the maximum and minimum of the weights of interactions between either of *P_i_*, *P_j_*, *P_k _*and a neighboring protein *P_r_*, respectively, where *r ≠ i*, *j*, *k *and (*i*, *r*) ∈ *E*, (*j*, *r*) ∈ *E*, or (*k*, *r*) ∈ *E*. It is considered that (F3), which is the maximum of the neighboring weights of a heterotrimer, should be lower than the weights of interactions in the heterotrimer. Consider the case that a protein *P_r _*interacts with two of proteins *P_i_*, *P_j_*, and *P_k_*, where *P_r _*is not any of *P_i_*, *P_j_*, and *P_k _*(see Figure 1). If the weights of both interactions are large, these proteins including *P_r _*may form a complex. We introduce the maximum of smaller weights of interactions with neighboring proteins *P_r _*denoted by (F5). (F6) and (F7) denote the maximum and the minimum of the numbers of domains contained in *P_i_*, *P_j_*, and *P_k_*, respectively. The number of domains in a protein complex is expected to be large because domains are considered as mediators of protein-protein interactions.

**Table 1:** Feature space mapping from three distinct proteins *P_i_*, *P_j_*, *P_k_*.

(F1)	$\underset{\left\{\left(p,q\right)\in E|p,q\in \left\{i,j,k\right\}\right\}}{\text{max}}w_{pq}$
(F2)	$\underset{\left\{\left(p,q\right)\in E|p,q\in \left\{i,j,k\right\}\right\}}{\text{min}}w_{pq}$
(F3)	$\underset{\left\{\left(p,r\right)\in E|p\in \left\{i,j,k\right\},r\notin \left\{i,j,k\right\}\right\}}{\text{max}}w_{pr}$
(F4)	$\underset{\left\{\left(p,r\right)\in E|p\in \left\{i,j,k\right\},r\notin \left\{i,j,k\right\}\right\}}{\text{min}}w_{pr}$
(F5)	$\underset{\left\{\left(p,r\right),\left(q,r\right)\in E|p,q\in \left\{i,j,k\right\},p\neq q,r\notin \left\{i,j,k\right\}\right\}}{\text{max}}\text{min}\left\{w_{pr},w_{qr}\right\}$
(F6)	max{# domains of *P_i_*, # domains of *P_j_*, # domains of *P_k_*}
(F7)	min{# domains of *P_i_*, # domains of *P_j_*, # domains of *P_k_*}

In addition to the extended features, we examine the domain composition kernel developed in our previous study [14]. We defined equivalence =*_d _*between two proteins *P_i _*and *P_j _*as the condition that *P_i _*consists of the same domains of *P_j _*, and defined equivalence =_*c *_between two sets ***x***_*i *_and ***x***_*j *_that consist of $\left\{P_{i_{1}},\cdot \cdot \cdot ,P_{i_{n}}\right\}$ and $\left\{P_{j_{1}},\cdot \cdot \cdot ,P_{j_{n}}\right\}$, respectively, as $\exists \;\sigma \in {픖}_{n}\forall k\left(P_{i_{k}}{=}_{d}P_{j_{\sigma \left(k\right)}}\right)$, where ${픖}_{n}$ denotes the symmetric group of degree *n *on the set {1, ⋯, *n*}. Then, the domain composition kernel *K_c _*was defined by

(2) $$K_{c}\left(x_{i},x_{j}\right)=\left\{\begin{array}{c} 1\;\;\;\;\left(\text{if}\;x_{i}{=}_{c}x_{j}\right),\\ 0\;\;\;\;(\text{otherwise)}\text{.} \end{array}\right)$$

### Two-phase learning approach

Our proposed methods take two-phase learning approach. The basic idea for designing our methods is that heterotrimeric protein complexes are not likely to share the same protein with other heterotrimeric protein complexes. We estimate model parameters of SVM using training data in the first phase, and predict whether or not the training data and the neighboring sets sharing at least one protein with the training data are heterotrimeric protein complexes, respectively. Then, the second phase predictor makes use of the discriminant values obtained by the first phase predictor. It is expected that the discriminant values for a target set of proteins and its neighboring set do not become large together if heterotrimeric protein complexes do not share the same protein.

**Figure 2:**
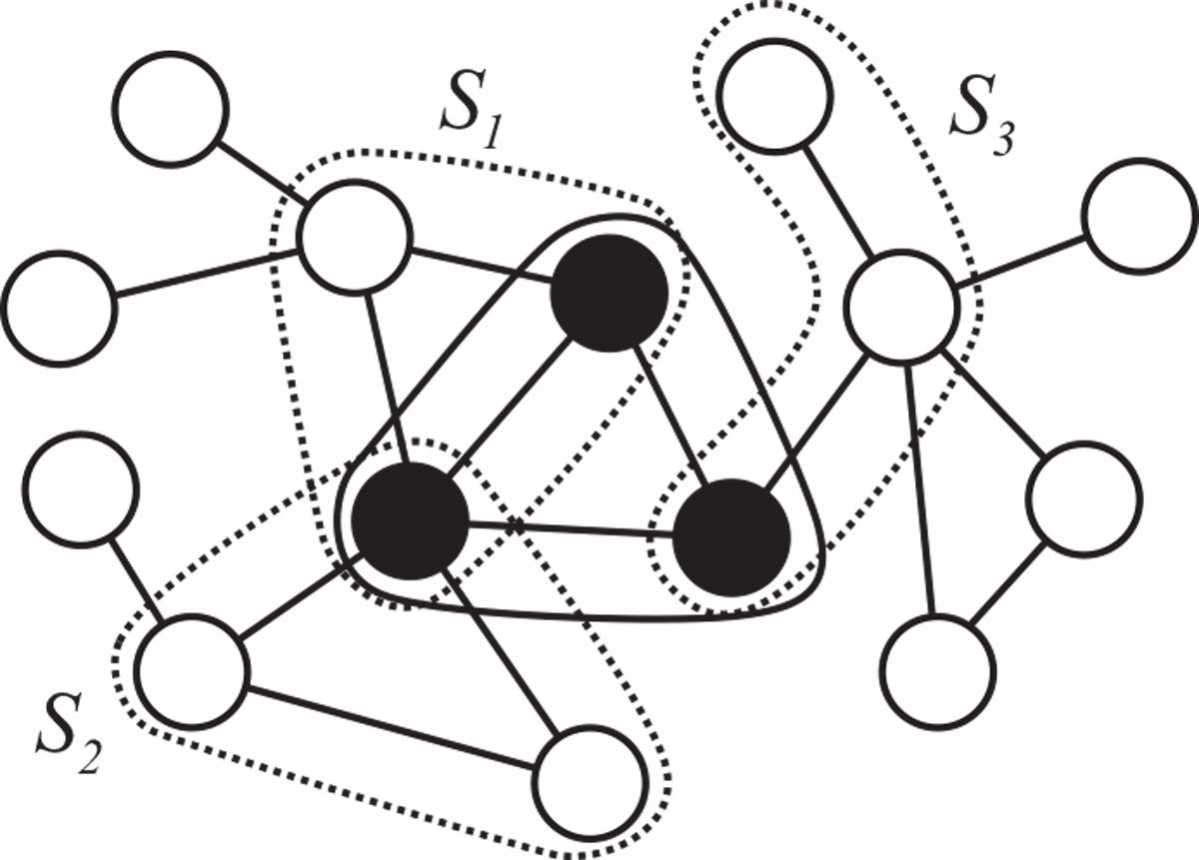
**Example of a subgraph including a focused set of proteins and neighboring sets of proteins**. Each neighboring set of three proteins shares at least one protein with the focused set (black circle). In this example, set *S*_1 _of three proteins shares two proteins with the focused set, and *S*_2_, *S*_3 _share one protein, respectively.

Suppose that the training data set comprises *N *sets ***x***_*i *_of three distinct proteins with the corresponding label *t_i _*∈ {-1, 1}. For each ***x***_*i*_, we calculate 7-dimensional feature vector ***f***^(1)^(***x***_*i*_) using (F1),…,(F7) shown in Table 1 and the kernel matrix whose (*i*, *j*)-th element is 〈***f***^(1)^(***x***_*i*_), ***f***^(1)^(***x***_*j*_)〉 + *αK_c_*(***x***_*i*_, ***x**_j_*), where *α *is a constant and $\left\langle \cdot ,\cdot \right\rangle$ denotes the inner product. Then, we obtain the model parameters in Eq. (1) by applying the SVM to the training data set. Let $N\left(x\right)$be all sets of three distinct proteins that are neighboring to ***x ***and connected in the protein-protein interaction network, where we call ***x***_*i *_a *neighboring *set to ***x***_*j *_if ***x***_*i *_and ***x***_*j *_share the same protein and ***x***_*i *_is not ***x***_*j *_(see Figure 2). For each ***x***_*i*_, we calculate the discriminant values *y*(***x***_*i*_) and *y*(***x***) for all $x\in N\left(x_{i}\right)$. Since the discriminant values may include outliers, by taking the averages of positive and negative discriminant values separately, we define four feature space mappings for ***x***_*i*_,

(3) $$f^{\left(2s\right)}\left(x_{i}\right)=y\left(x_{i}\right),$$

(4) $$f^{\left(2p\right)}\left(x_{i}\right)=\frac{1}{\left|\left\{x\in N\left(x_{i}\right)|y\left(x\right)>0\right\}\right|}\underset{\left\{x\in N\left(x_{i}\right)|y\left(x\right)\;>\;0\right\}}{\sum }y\left(x\right),$$

(5) $$f^{\left(2n\right)}\left(x_{i}\right)=\frac{1}{\left|\left\{x\in N\left(x_{i}\right)|y\left(x\right)<0\right\}\right|}\underset{\left\{x\in N\left(x_{i}\right)|y\left(x\right)\;<\;0\right\}}{\sum }y\left(x\right),$$

(6) $$f^{\left(2a\right)}\left(x_{i}\right)=\frac{1}{\left|N\left(x_{i}\right)\right|}\underset{x\in N\left(x_{i}\right)}{\sum }y\left(x\right),$$

where |*S*| denotes the number of elements in the set *S*. Here, we define *f *^(2*p*)^(***x***_*i*_) = 0 (*f *^(2*n*)^(***x***_*i*_) = 0, *f *^(2*a*)^(***x***_*i*_) = 0) if $|\left\{x\in N\left(x_{i}\right)|y\left(x\right)>0\right\}|=0\left(|\left\{x\in N\left(x_{i}\right)|y\left(x\right)<0\right\}|=0,|N\left(x_{i}\right)|=0\right)$. We compose 11-dimensional feature vector ***f ***^(2)^(***x***_*i*_) using ***f ***^(1)^, *f *^(2*s*)^, *f *^(2*p*)^, *f *^(2*n*) ^and *f *^(2*a*)^, calculate the kernel matrix with the (*i*, *j*)-th element 〈***f ***^(2)^(***x***_*i*_), ***f ***^(2)^(***x***_*j *_)〉 + *αK_c_*(***x***_*i*_, ***x**_j_*), and we apply some supervised learning method. It should be noted that our methods use only training data to estimate model parameters. For test data ***x***, we calculate 〈***f ***^(2)^(***x***_*i*_), ***f ***^(2)^(***x***)〉 + *αK_c_*(***x***_*i*_, ***x***) for training data ***x***_i_, and determine whether or not ***x ***is a heterotrimeric protein complex according to the second classifier.

## Computational experiments

### Data and implementation

To evaluate our proposed methods, we performed computational experiments and compared them with the existing method NWE [5]. We used the WI-PHI database [1] containing 49607 interacting protein pairs except self interactions as input weights of interactions, which is available at the supporting information web page of the paper. The weights were obtained from high-throughput yeast two-hybrid data [18,19] and several biological databases such as BioGRID [2] and BIND [20] by using a log-likelihood score (LLS) to each dataset and the socioaffinity (SA) index [21] that measures the log-odds score of the number of times that two proteins are observed to interact to the expectation value from the dataset.

We prepared datasets using heterotrimeric protein complexes in CYC2008 protein complex catalogue [12], which contains 87 heterotrimeric protein complexes, and is available at http://wodaklab.org/cyc2008/. We restricted positive and negative examples to sets of three distinct proteins that form a single connected component in the input protein-protein interaction network. Thus, 7 heterotrimers were eliminated, and we used 80 heterotrimers as positive examples. For negative examples, we extracted 32647 sets of three proteins included in protein complexes with size more than three of CYC2008, and we selected uniquely at random 100 examples from the sets because our methods require many neighboring sets of three proteins for an example in the second phase. It is considered that negative examples selected from such sets are more difficult to be classified than those selected from all sets of three proteins except heterotrimers.

For NWE, we set some options related with the size of complexes so that NWE output protein complexes with size two or more from the WI-PHI protein-protein interaction network in the same way as [13], and extracted only protein complexes with size three from the result.

For measuring the performance, we used accuracy, precision, recall, and F-measure defined by

(7) $$accuracy=\frac{TP+TN}{TP+TN+FP+FN},$$

(8) $$precision=\frac{TP}{TP+FP},$$

(9) $$recall=\frac{TP}{TP+FN},$$

(10) $$F\text{ - }measure=\frac{2\;\cdot \;precision\;\cdot \;recall}{precision+recall},$$

where *TP*, *FP*, and *FN *mean the number of true positive, false positive, false negative examples, respectively.

We used 'libsvm' (version 3.11) [22] and 'SparseBayes' package (version 2.0) [23] as implementations of SVM and RVM, respectively.

### Results

We performed 10-fold cross-validation, and took the average of accuracy, precision, recall, and F-measure. Furthermore, we repeated this procedure 10 times for other datasets with randomly selected negative examples, and took the average. Table 2 shows the results on the average of accuracy, precision, recall, and F-measure by our proposed methods and NWE. 'SVM+SVM' and 'SVM+RVM' denote two-phase methods using SVM and RVM as the second classifier, respectively. 'SVM' denotes usual SVM using only features ***f ***^(1)^. *α *denotes the coefficient of the domain composition kernel *K_c_*. We examined *α *= 0.5 because the case was best for prediction of heterodimeric protein complexes in our previous study [14]. NWE predicted 54 protein complexes with size three from the WI-PHI protein-protein interaction network, and 19 of them were actual heterotrimeric protein complexes in the CYC2008 protein complex catalogue. We can see from the table that the F-measure by SVM+SVM, SVM+RVM, SVM for both *α *= 0, and 0.5 were higher than those by NWE, respectively. Furthermore, the accuracy and F-measure by the two-phase method SVM+SVM were higher than those by usual SVM with ***f ***^(1)^, respectively. The accuracy and F-measure by SVM+RVM, however, were lower than those by SVM, respectively. It implies that RVMs may be less useful than SVMs for these problems that SVMs can be applied. Thus, the results suggest that our proposed methods SVM+SVM, SVM+RVM, and SVM outperform the existing method NWE. The results also suggest the usefulness of the second phase.

**Table 2:** Results on the average of accuracy, precision, recall, and F-measure by our proposed methods and NWE.

	SVM+SVM		SVM+RVM		SVM		NWE
		
*α*	0	0.5	0	0.5	0	0.5	
accuracy	0.885	**0.907**	0.810	0.853	0.861	0.876	-
precision	**0.936**	0.869	0.847	0.899	0.909	0.873	0.352
recall	0.840	**0.926**	0.770	0.766	0.819	0.862	0.218
F-measure	0.880	**0.891**	0.767	0.810	0.854	0.862	0.270

'SVM+SVM' and 'SVM+RVM' denote two-phase methods using SVM and RVM as the second classifier, respectively. 'SVM' denotes usual SVM using only features ***f***^(1)^. *α *denotes the coefficient of the domain composition kernel *K*_*c*_. Note that the accuracy is not defined for NWE because it is unsupervised, and predict protein complexes of various sizes. The precision and recall for NWE were calculated as *TP *divided by the numbers of predicted and known heterotrimers, respectively.

## Conclusions

We proposed prediction methods by two-phase learning for heterotrimeric protein complexes. In the methods, we extended the feature space mappings in our previous study for prediction of heterodimeric protein complexes, and made use of the discriminant function for neighboring sets of three proteins. To validate our proposed methods, we performed 10-fold cross-validation computational experiments. The results suggest that our two-phase prediction methods and SVM with the extended features outperform the existing method NWE, which was reported to outperform many other existing methods such as MCL, MCODE, DPClus, CMC, COACH, RRW, and PPSampler, although our methods are limited to prediction of heterotrimeric protein complexes. For further evaluation, we would like to perform computational experiments for other datasets if such data become available.

We have some possibility to further improve the prediction accuracy. For instance, we can use sequence information for designing feature space mappings as well as domains contained in proteins. In addition, we can introduce some probabilistic model such as conditional and Markov random fields to neighboring sets of three proteins although in this paper we considered kernels between neighboring sets.

## Competing interests

The authors declare that they have no competing interests.

## Authors' contributions

PR and MH developed and implemented the methods. MH drafted the manuscript. OM and TA participated in the discussions during the development of the methods and helped draft the manuscript. All authors read and approved the final manuscript.

## Declarations

The publication costs for this article were funded by JSPS, Japan (Grants-in-Aid #22240009).

This article has been published as part of *BMC Bioinformatics *Volume 15 Supplement 2, 2014: Selected articles from the Twelfth Asia Pacific Bioinformatics Conference (APBC 2014): Bioinformatics. The full contents of the supplement are available online at http://www.biomedcentral.com/bmcbioinformatics/supplements/15/S2.
